# A bi-annotated Malay-English code-switching (Manglish) dataset of X posts for biological gender identification and authorship attribution

**DOI:** 10.1016/j.dib.2024.110034

**Published:** 2024-01-08

**Authors:** Ruhaila Maskat, Norazmiera Ayunie Azman, Nur Shaheera Shastera Nulizairos, Nurul Athirah Zahidin, Adibah Humairah Mahadi, Siti Rubaya Norshamsul, Mohd Mukhlis Mohd Sharif, Hairulnizam Mahdin

**Affiliations:** a College of Computing, Informatics and Mathematics of Universiti Teknologi MARA Shah Alam, 40450, Selangor, Malaysia; b Faculty of Computer Science and Information Technology, Universiti Tun Hussein Onn Malaysia, 86400 Parit Raja, Batu Pahat, Johor, Malaysia

**Keywords:** Biological gender identification, Authorship attribution, Code-switching, Malay-English, Manglish, NLP, Text analytics

## Abstract

Low-resource languages, like Malay, face the threat of extinction when linguistic resources become scarce. This paper addresses the scarcity issue by contributing to the inventory of low-resource languages, specifically focusing on Malay-English, known as Manglish. Manglish speakers are primarily located in Malaysia, Indonesia, Brunei, and Singapore. As global adoption of second languages and social media usage increases, language code-switching, such as Spanglish and Chinglish, becomes more prevalent. In the case of Malay-English, this phenomenon is termed Manglish. To enhance the status of the Malay language and its transition out of the low-resource category, this unique text corpus, with binary annotations for biological gender and anonymized author identities is presented. This bi-annotated dataset offers valuable applications for various fields, including the investigation of cyberbullying, combating gender bias, and providing targeted recommendations for gender-specific products. This corpus can be used with either of the annotations or their composite. The dataset comprises of posts from 50 Malaysian public figures, equally split between biological males and females. The dataset contains a total of 709,012 raw X posts (formerly Twitter), with a relatively balanced distribution of 53.72% from biological female authors and 46.28% from biological male authors. Twitter API was used to scrape the posts. After pre-processing, the total posts reduced to 650,409 posts, widening the gap between the genders with the 56.88% for biological female and 43.12% for biological male. This dataset is a valuable resource for researchers in the field of Malay-English code-switching Natural Language Processing (NLP) and can be used to train or enhance existing and future Manglish language transformers.

Specifications Table

**Table:** 

Subject	Data Science: Big Data Analytics, Data Mining and Machine Learning.
Specific subject area	Attributing the authorship of a Manglish X post (formerly Twitter) and discerning the biological gender of the author, constrained within a binary categorization (male and female).
Data format	Raw, Pre-processed
Type of data	.csv file
Data collection	Given the inherent low-resource characteristics of Manglish X posts, stringent selection criteria were devised. Initially, the sample was confined to prominent Malaysian public figures, encompassing influencers, politicians, academicians, and artists, with biological genders as male or female. Additionally, a minimum threshold of 1,000 was set for the total number of posts, irrespective of the temporal distribution for each author. Furthermore, a prerequisite of approximately 60% or more of the generated posts was that they be composed in Manglish. Notably, images were excluded from the dataset, and the raw version retained elements such as emojis and reposts.
Data source location	X (Formerly Twitter) site.
Data accessibility	Repository name: Open Science Framework (OSF) Direct URL to data: https://osf.io/um8k7/?view_only=81fe6e164e994c87851f0845c36d41da DOI:10.17605/OSF.IO/UM8K7
Related research article	Currently in writing, not yet published.

## 1 Value of the Data

• Scholars within the field of Manglish Natural Language Processing (NLP) may leverage this dataset to formulate innovative methodologies pertaining to the pre-processing and feature selection of text data sourced from social media platforms.
• This dataset constitutes a substantial contribution to the ongoing endeavors aimed at addressing the persistent challenges posed by the low-resource nature of Manglish social media text, particularly within the research community.
• The annotated characteristics of this dataset render it a benchmark for the training and evaluation of extant or emerging machine learning and deep learning models, aimed at the discernment of male and female genders in Malay/Manglish posts and the discrimination between authors.
• This dataset can serve as a valuable resource for training both novel and pre-existing Manglish language transformers.
• This dataset lays the foundation for a diverse spectrum of applications in both biological gender identification and authorship attribution. In the context of gender identification, it facilitates the development of personalized content and recommendations, fosters an in-depth comprehension of consumer behavior, preferences, and shopping patterns, reinforces authentication and security protocols through the validation of biological gender, enables tailored medical advice, and promotes scholarly investigations into gender-related phenomena, encompassing gender bias, gender roles, and social dynamics. In the domain of authorship attribution, it empowers the realms of plagiarism detection, facilitates criminal investigations involving anonymous texts, resolves disputes concerning the authorship of contested works, verifies the authenticity of legal documents, contracts, and wills, aids in identifying individuals responsible for instances of hate speech, harassment, or harmful behaviors, and offers valuable insights into the attribution of authorship within historical documents.
• Diverse algorithms may be employed for the prediction of distinct models within the domain of biological gender classification and/or authorship attribution.

## 2 Background

Datasets on Manglish social media text that are publicly accessible are scarce. Most datasets are proprietary; thus, experiments are not easily replicable. This dataset aims to in part address this in the area of biological gender identification and author attribution. At the time of writing, no dataset of this likeness in Malay/Manglish is available for interested researchers to utilize as a benchmark. We hope this effort can narrow this scarcity gap. The task of identifying biological gender from social media text is useful in multiple areas of research. To describe a few [1,2]; it facilitates the development of personalized content and recommendations, fosters an in-depth comprehension of consumer behavior, preferences, and shopping patterns, reinforces authentication and security protocols through the validation of biological gender, enables tailored medical advice, and promotes scholarly investigations into biological gender-related phenomena, encompassing gender bias, gender roles, and social dynamics. In the domain of authorship attribution, it empowers the realms of plagiarism detection, facilitates criminal investigations involving anonymous texts, resolves disputes concerning the authorship of contested works, verifies the authenticity of legal documents, contracts, and wills, aids in identifying individuals responsible for instances of hate speech, harassment, or harmful behaviors, and offers valuable insights into the attribution of authorship within historical documents [3], [4], [5]. To ensure thorough learning for machine learning algorithms across different author and biological gender profiles, we balanced the dataset. This involved selecting 25 biological male and 25 biological female authors, each providing a minimum of 1,000 Manglish X posts. However, achieving a similar balance across authors' varied backgrounds (e.g., academicians or politicians) posed challenges due to the primary factor of availability. Despite these hurdles, we believe this dataset is as balanced as realistically possible.

## 3 Data Description

The dataset under examination has been segregated into two .csv files, denoted as “Raw.csv” and “Preprocessed.csv”. Both files encompass posts that are curated with labels such as “Female,” “Male,” and identifiers from “Author1” through “Author50.” The attributes inherent to the dataset are succinctly detailed in Table 1, encompassing biological gender, anonymized author id, and the post content. Furthermore, Table 2 provides illustrative examples of posts contained within the dataset. We translate the Manglish post directly to English to give readers understanding of the form of code-switching that occurred.

**Table 1:** Description of attributes constituting the dataset.

Attribute	Description
Gender	Male or Female labels
Author	Author1, Author2, … Author50 labels
XPost	The content of the post

**Table 2:** Example X posts.

Malay-English	English translation	Gender	Author
Bestnya ada pintu suka hati	How great, there is a door, am happy	Male	Author1
And so it begins. orang shortlisted dan di panggil untuk live audition kat @ERAdotje.	And so, it begins. People who were shortlisted and called for a live audition at @ERAdotje.	Male	Author2
Tell me which one you guys nak beli?!	Tell me which one you guys wants to buy?!	Female	Author3
I nak bg giveaway 1 love letter perfume utk org KL/selangor	I want to giveaway one love letter perfume for people in KL/Selangor	Female	Author4

### 3.1 Raw dataset

**Fig. 1:**
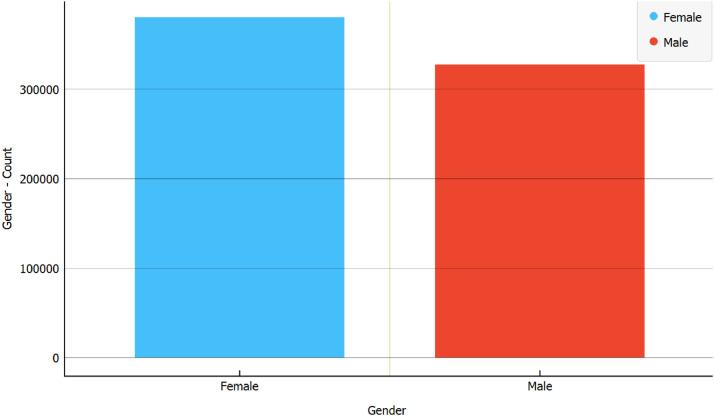
Number of biological female and male posts.

Within the raw dataset, there were 380,865 posts authored by biological females, representing 53.72% of the total, while 328,147 posts, making up 46.28% of the complete dataset, were authored by biological males. This cumulative total amounts to 709,012 posts. This distribution demonstrates a fairly balanced representation of gender within the dataset. Refer Fig. 1.

**Fig. 2:**
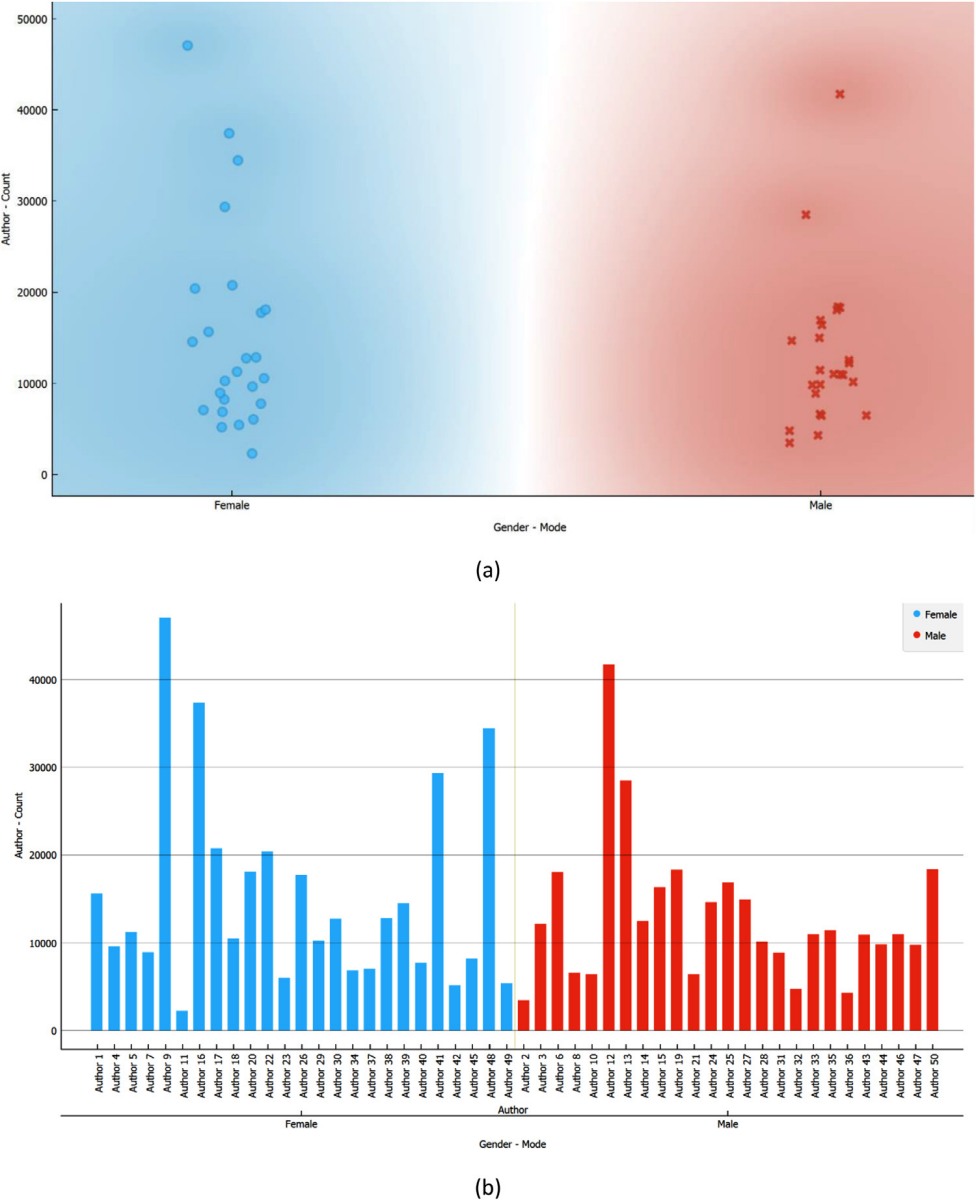
Distribution of authors’ posts grouped by their gender in the raw dataset.

In Fig. 2, we present the distribution of posts for all authors. In subfigure (a), a scatterplot is employed to depict that the majority of total posts fall within the range of 20,000 or less for both biological genders. Subfigure (b) provides the anonymized IDs of the authors, with Author9 being the most prolific, closely followed by Author12. Conversely, Author11 and Author2 contributed the least number of posts. This equitable distribution of posts between biological genders serves to mitigate potential biases in the dataset.

**Fig. 3:**
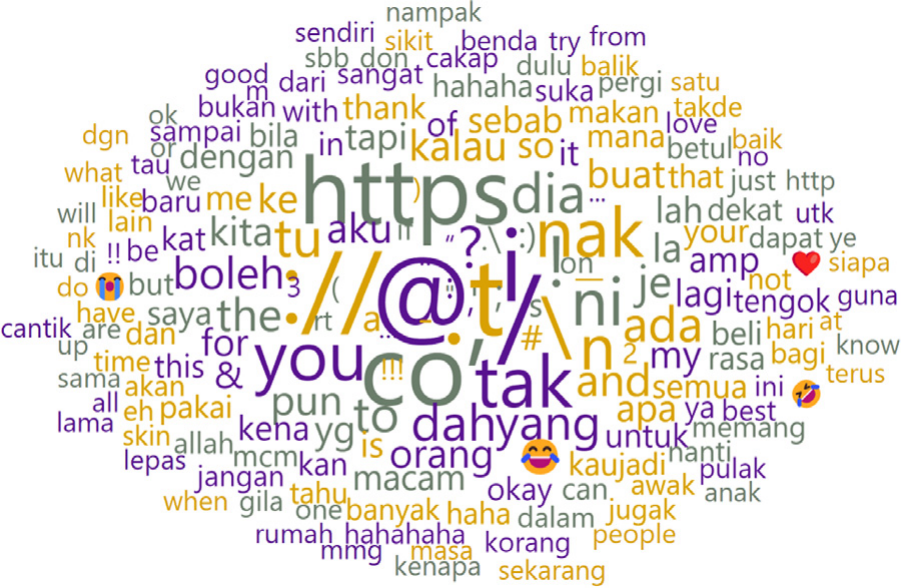
Word cloud of biological female writers.

In Fig. 3, we have employed the Orange Data Mining tool to generate a word cloud illustrating the most frequent words, symbols, and emojis authored by biological female individuals. The size of each word or symbol in the cloud corresponds to its popularity in terms of usage frequency. Furthermore, in Table 3, we have cataloged the most prevalent emojis.

**Table 3:** Emojis frequently used by biologically female authors.

No.	Emoji	Description (emojitranslate.com)	Frequency
1		Laugh out loud (LOL)	15,736
2		Loudly crying	6,187
3		Heart	5,995
4		Rolling on the floor laughing	5,230

**Fig. 4:**
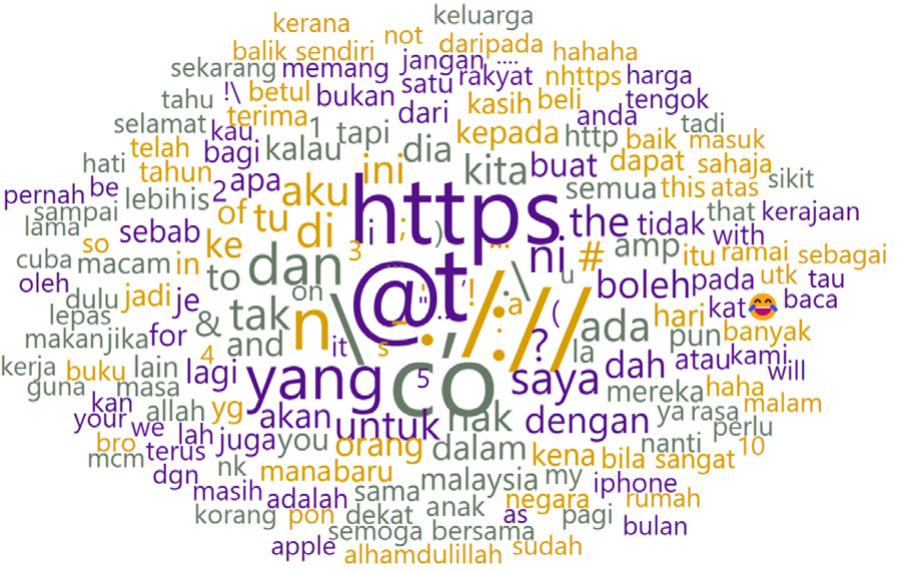
Word cloud of biologically male writers of raw dataset.

In Fig. 4, a word cloud visually represents the most frequently used words and symbols by biological males. Table 4 complements this depiction by highlighting the Laugh Out Loud (LOL) emoji as the most frequently used, occurring 5,011 times.

**Table 4:** Emoji frequently used by biologically male authors in the raw dataset.

No.	Emoji	Description (emojitranslate.com)	Frequency
1		Laugh out loud (LOL)	5,011

### 3.2 Pre-processed dataset

**Fig. 5:**
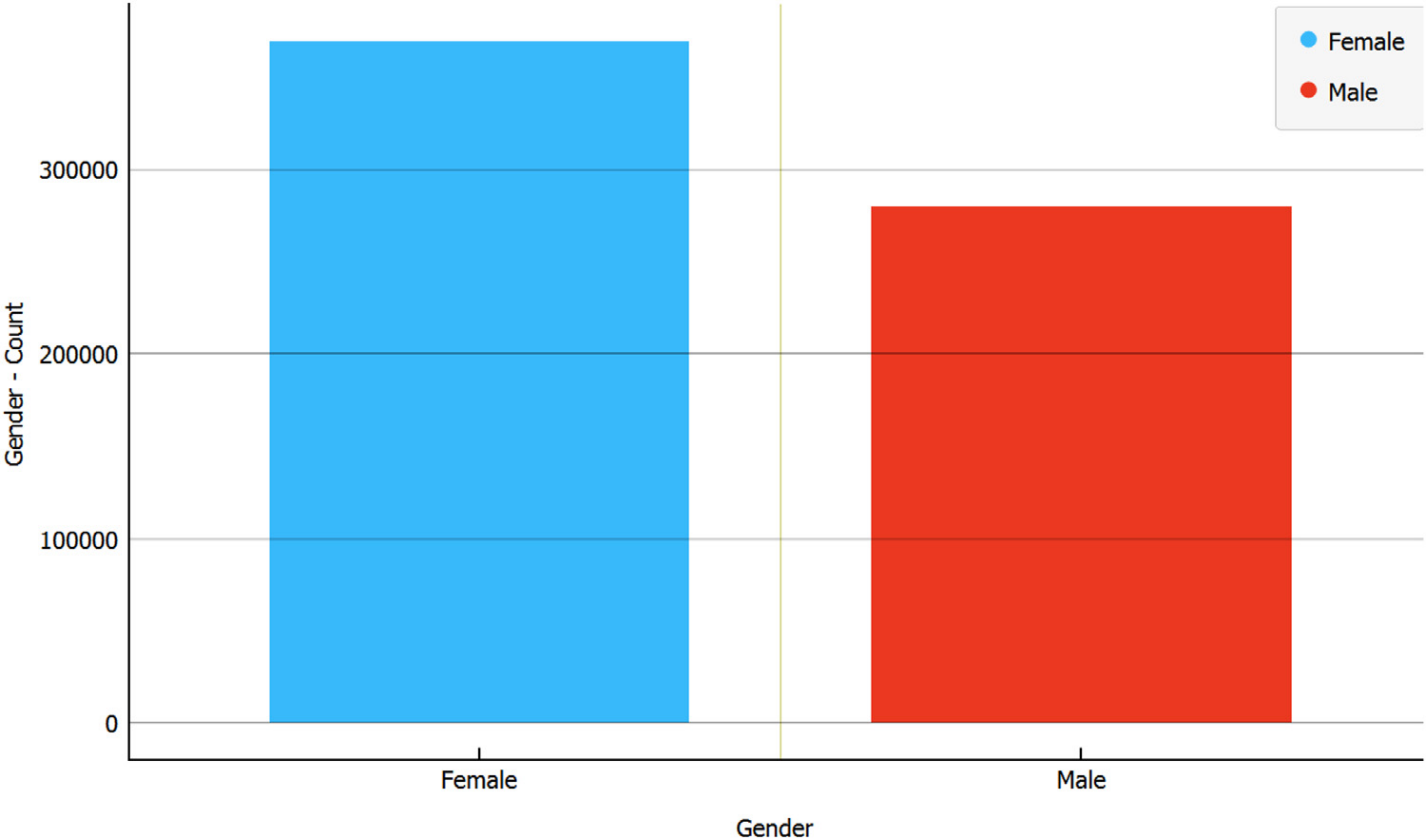
Total of biological male and female posts in the pre-processed dataset.

Following the pre-processing stage, the dataset was refined to encompass a total of 650,409 posts. Among these, posts authored by individuals who are biologically female amounted to 369,925, constituting 56.88% of the total. Biologically male authors contributed 280,484 posts, accounting for 43.12% of the dataset. It is noteworthy that the dataset maintains a relatively balanced distribution, consistent with its state prior to pre-processing. Fig. 5 visualizes the comparison.

**Fig. 6:**
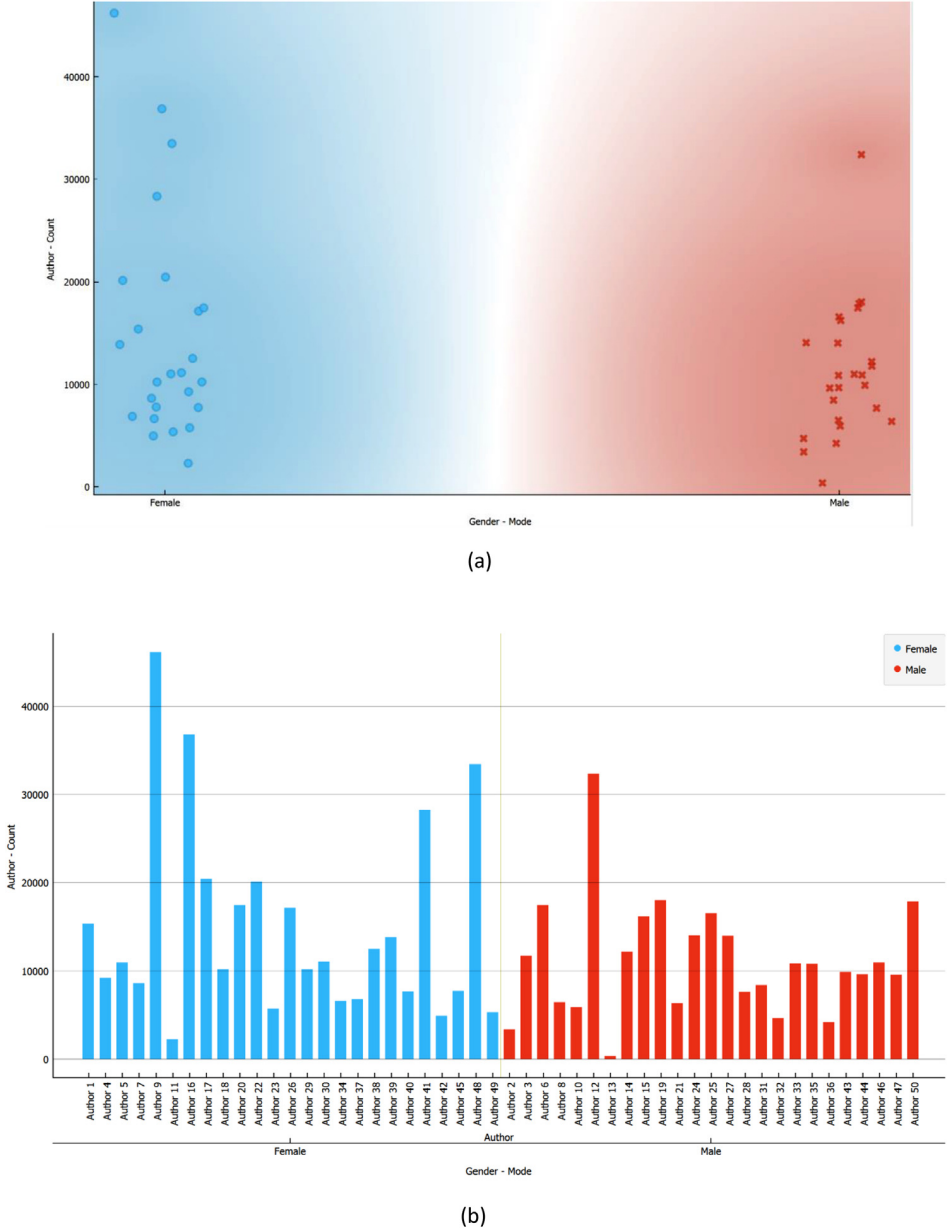
Distribution of authors by their gender in the pre-processed dataset.

The gender distribution of authors is visualized in Fig. 6, revealing a pattern akin to the raw dataset. In subfigure (a), the scatterplot illustrates a concentration of posts per author around the 20,000 and below mark for both male and female authors. Subfigure (b) indicates that the ranking of female authors in terms of the number of posts remained largely unchanged after pre-processing. In contrast, certain male authors exhibited a notable reduction, with Author13 experiencing the most substantial decrease, transitioning from the second-highest post count to the least. Another male author, Author28, also underwent a considerable reduction, though not as pronounced as Author13. Author43 represents the last male author to undergo a reduction due to pre-processing, but subtler.

**Fig. 7:**
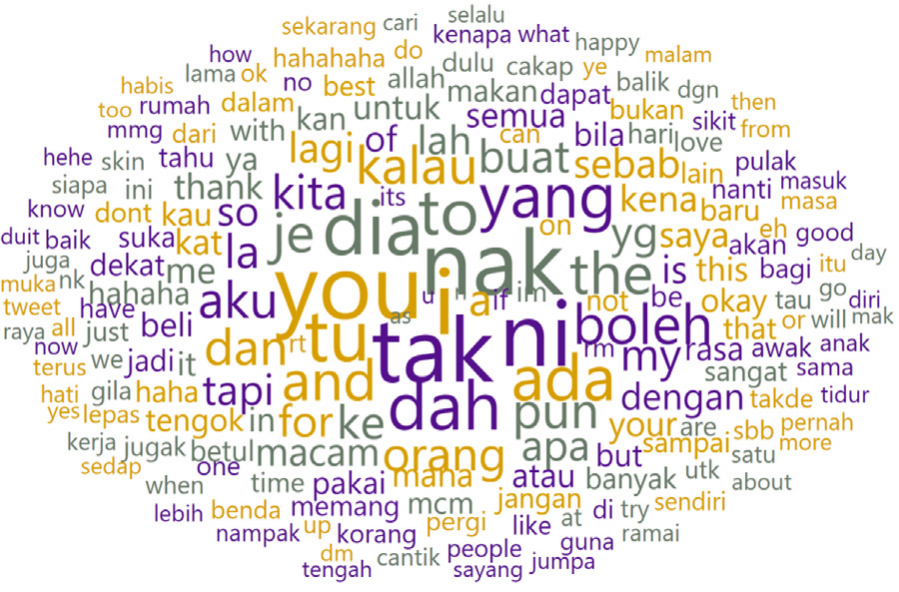
Word cloud of biological female writers after pre-processing.

Utilizing the Orange Data Mining tool once more, we generated a word cloud depicting the most frequently used words by individuals identified as biologically female. Fig. 7 illustrates the word cloud of posts by female authors and Table 5 supplements it by showcasing the frequency of the top ten most commonly used words. Given the code-switching characteristics inherent in the dataset, we have compiled and translated these words into English for the understanding of interested researchers.

**Table 5:** Ten most frequent words by biologically female authors.

No.	Manglish	English translation	Frequency
1	I	I	90,737
2	Tak	no, not	63,472
3	Nak	want	58,316
4	You	you	54,683
5	Ni	this	51,864
6	Dia	him, her	40,860
7	Tu	there, that	38,774
8	Dah	already	35,633
9	To	to	33,790
10	Ada	have, got	33,410

**Fig. 8:**
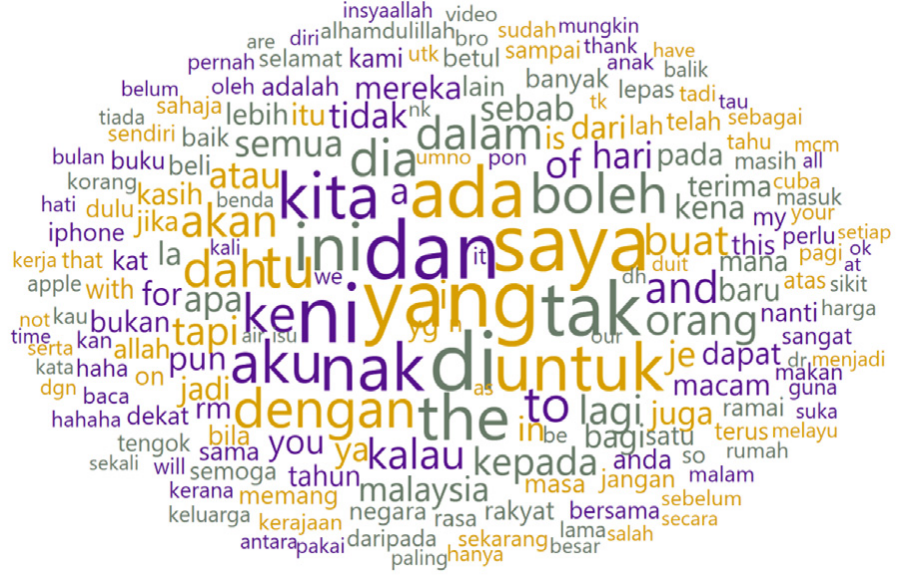
Word cloud of biological male writers after pre-processing.

We display the word cloud for biological male authors in Fig. 8 and the top ten frequently used words in Table 6. Similarly, the words underwent direct translation to English for better understanding.

**Table 6:** Ten most frequent words by biologically male authors.

No.	Manglish	English translation	Frequency
1	Yang	which, that, darling	63,749
2	Dan	and	60,634
3	Di	at	36,612
4	Saya	I, me	36,374
5	Ni	this	35,790
6	Tak	no, not	32,130
7	Ada	have, got	30,278
8	Nak	want	28,026
9	Ini	this	27,908
10	Untuk	for	27,875

## 4 Experimental Design, Materials and Methods

**Fig. 9:**
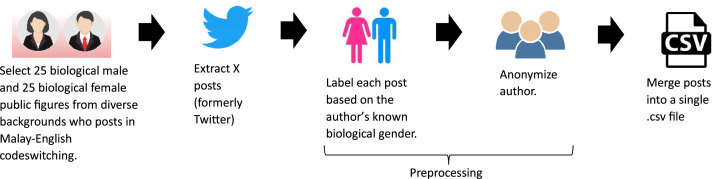
Processes involved in dataset formation.

The construction of the dataset required the execution of five distinct processes, as visually illustrated in Fig. 9. This sequence commenced with the initial candidate selection, followed by the subsequent extraction of X posts, the annotation of biological gender labels, author identity anonymization, and concluded with the consolidation of all posts.

### 4.1 Selection of candidates

Given the prevalence of pseudonymous identities, a secure and reliable method for dataset construction involved the selection of 25 biological male and 25 biological female candidates who are public figures. Public figures, in contrast to random public X accounts, offer a more certain determination of biological gender, thereby reducing the risk of false gender representation, as has been observed in numerous cases of online love scams. These public figures are individuals known for their integrity, thus lacking a reasonable motive to misrepresent their biological gender. They encompass a diverse range of roles, including influencers (20 individuals), politicians (8 individuals), celebrities (5 individuals), entrepreneurs (11 individuals), writers (4 individuals), and academicians (2 individuals).

In addition to this criterion, the dataset inclusion relied on a substantial total post count, with a threshold set at 1,000 posts or more, regardless of the time frame. Furthermore, the determination of eligibility required that at least 60 percent of the posts be composed in Malay-English codeswitching, with this determination made in real-time. Importantly, no images were downloaded alongside the dataset for the purpose of maintaining data integrity and consistency.

### 4.2 X posts extraction

Upon the culmination of the candidate selection procedure, we retrieved X posts utilizing the Twitter API. This data extraction activity was conducted within the time span of November 28 to December 2, 2022, and it was executed by a team of six university students who were working under thesis supervision. Consequently, the acquired data was organized and stored in distinct .csv files, with one file allocated to each respective author.

### 4.3 Pre-processing of data

The pre-processing of the extracted raw data was performed using Jupyter Notebook, which involves importing essential Python libraries such as *pandas, re, html, numpy*, and *os*. Initially, the raw data was loaded into a Pandas DataFrame using the *pd* library. Regular expressions were used to clean and manipulate text data, and the *html* library was used to handle and encode the entities in the dataset. The *numpy* library supported various operations. Additionally, the *os* library was used for file and directory management. The following shows the steps taken.

#### 4.3.1 Step 1: data loading

The raw data was loaded into a dataframe using the Pandas *read_csv* function to read CSV format data.

#### 4.3.2 Step 2: attributes selection

While the raw data initially comprised 79 attributes, our research specifically required the utilization of only three attributes. Consequently, we chose to focus on the following attributes: 'author.username', 'text', and 'references_tweet.retweet.id'.

#### 4.3.3 Step 3: repost removal

The extracted data consisted of both posts written by authors and posts that were reposted by other authors. Hence, repost removal needed to be done. Consequently, a repost identification and removal process were essential. To distinguish reposted content, we relied on the 'reference_tweets.retweeted.id' attribute. If this attribute contained a distinct value, the corresponding data was classified as reposted content. Conversely, when the attribute had a missing value, it was categorized as non-reposted content. A filtering step was implemented to retain only those rows where the 'referenced_tweets.retweeted.id' column contained missing values (NaN). In the subsequent phase, the 'referenced_tweets.retweeted.id' column was removed from the DataFrame, resulting in a dataset exclusively containing non-reposted text.

#### 4.3.4 Step 4: gender attributes added

A new column, denoted as 'Gender', was introduced to the dataframe. For each row, the value within this 'Gender' column was determined and assigned as either 'Male' or 'Female,' contingent upon the biological gender of the respective author.

#### 4.3.5 Step 5: rename the column

Two columns within the DataFrame underwent a renaming process. Specifically, the 'text' column was relabeled as 'Text', and the 'author.username' column was renamed as 'Author'.

#### 4.3.6 Step 6: arrange the columns

This step was undertaken to establish a standardized column order for each author, commencing with 'Author', followed by 'Gender', and concluding with 'Text'.

#### 4.3.7 Step 7: author anonymization

In order to protect the anonymity of the authors, the 'Author' column, which contained the authors' usernames, underwent a transformation where each author's username, such as “John Smith”, was substituted with alternative identifiers, e.g., 'Author1′. This anonymization process was consistently applied to all authors, extending up to Author 50. This column is designated as the target variable for the authorship attribution task. However, the analysis raises two potential concerns relating to bias or inadvertent data exposure. These concerns involve the presence of an author's username within a post and the utilization of pronouns. Regarding the former, instances of an author's username in the posts were not anonymized to maintain essential contextual information required for analysis, as these references were intertwined with numerous mentions of other public figures. Concerning the latter, in the Malay language, singular and plural pronouns lack gender specification. For instance, the singular pronoun 'dia' could refer to either 'he' or 'she', while the plural pronoun 'mereka' translates to 'they'. Consequently, anonymization of pronouns is deemed unnecessary for researchers.

#### 4.3.8 Step 8: remove user mentions and emojis

A function, denoted as 'clean_text', was introduced to facilitate the text cleaning process by eliminating user mentions (e.g., '@username') and emojis. This function conducts a validation check to ensure the input is a string, then proceeds to execute the cleaning operations, ultimately yielding the sanitized text. Subsequently, the function was employed on a DataFrame column titled 'Text', with the resultant cleaned text being stored in a newly created column known as 'Text1′ within the same DataFrame.

#### 4.3.9 Step 9: remove URLs

An auxiliary function, designated as 'remove_links', was implemented to eliminate URLs within the 'Text1′ column, a step carried out subsequent to the 'clean_text' procedure. The result of this operation is subsequently saved in a newly established column named 'Text1′ within the same DataFrame.

#### 4.3.10 Step 10: decoding HTML entities

The introduction of a function titled 'decode_html_entities' was pivotal in the research process. This function was designed to decode HTML entities, such as '&amp', within the text. It commences by verifying the input as a string, employs the 'html.unescape' function to decode HTML entities, and subsequently archives the outcomes within a fresh column named 'Text3′.

#### 4.3.11 Step 11: remove newlines character

The 'remove_newlines' function was harnessed to eliminate newline characters from the text. It systematically replaced distinct newline sequences, including .nn, .nnn, .n, and nn, with spaces. The outcome of this operation was subsequently archived in a new column labeled 'Text4′.

#### 4.3.12 Step 12: remove leading whitespaces

The 'remove_whitespace' function was instrumental in streamlining the data. It targeted the removal of redundant leading whitespace from the text in each row. To accomplish this, the function commenced by verifying whether the input was in string format, and then proceeded to utilize the strip function for whitespace removal. The resulting cleaned text was then stored in a newly designated column named 'Text5′.

#### 4.3.13 Step 13: convert text to lowercase

To standardize the text, a transformation to lowercase is imperative. Therefore, the 'to_lowercase' function is implemented to facilitate the conversion of the text to its lowercase equivalent. The outcome of this operation is preserved in a fresh column labeled 'Text6′.

#### 4.3.14 Step 14: remove punctuation marks and symbols

The 'remove_punctuation' function is employed to eradicate a range of punctuation marks, commas, and symbols, including [.,!?():{}[]='"\∼#$%^()-_+∼`;|&<>], from the text. This operation is executed through the use of regular expressions. The resulting purified text is then archived in a freshly generated column designated as 'Text7′.

#### 4.3.15 Step 15: replace symbols with Malay language equivalents

Two symbols, namely '&' and '/', were substituted with 'dan' and 'atau', respectively. This adaptation was necessitated by the prevalence of Malay language context, as it plays a crucial role in conveying the textual meaning. ‘Dan’ is ‘and’ while ‘atau’ is ‘or’ in English.

#### 4.3.16 Step 16: selecting and renaming columns

Following the processing sequence, the 'Text8′ column uniquely undergoes all pre-processing procedures. Consequently, 'Text8′ is designated for inclusion in a fresh data frame, alongside the 'Author' and 'Gender' columns. This integration involves the renaming of the 'Text8′ column in 'df5′ to 'Text', effectively altering the column's nomenclature.

#### 4.3.17 Step 17: handling whitespace-only rows in text column

Within the newly created 'Text' column, certain rows exclusively consisted of whitespace characters; however, these rows were not initially identified as missing values. Consequently, the code took the initiative to set these whitespace-only values in the 'Text' column of 'df5′ as NaN. Subsequently, a command was executed to eliminate rows within 'df5′ in which the 'Text' column contained NaN values, thus efficiently removing entries with empty or whitespace-only text content.

#### 4.3.18 Step 18: saving cleaned data to CSV

The resultant 'df5′ DataFrame holds sanitized data that has been stored in the designated CSV file. This identical procedure is replicated for the remaining 49 authors.

#### 4.3.19 Step 19: combining author files into a single CSV

Utilizing the *os* library, our code amalgamates the individual CSV files of each author into a unified CSV file. This process involves the reading of multiple CSV files from a designated folder, merging them into a singular DataFrame, and the assignment of column headers to establish a consolidated dataset.

## Limitations

In an effort to mitigate potential biases stemming from authorship and biological gender, we endeavored to achieve a balanced distribution of posts across the selected X accounts. However, due to Malay language being low-resourced, we encountered limitations in the selection of X accounts that prominently featured Malay-English code-switching. Furthermore, we faced challenges related to the total post count per author, with some accounts predominantly featuring code-switching text falling short of the required post threshold. Additionally, we observed an abundance of accounts meeting our selection criteria among biologically male authors, in contrast to those from biologically female authors. It is noteworthy that, based on our experience, female authors tended to prefer full English language usage over Manglish in their posts.

## Ethics Statement

Data for this study was obtained through web scraping using the Twitter API from publicly available accounts, ensuring compliance with scraping policies. Anonymization measures were applied to protect author privacy in line with legal and ethical standards governing social media data research. Notably, named entities and tagged accounts were retained to maintain contextual relevance for the NLP task. The dataset, originating from X (formerly Twitter), serves the purpose of enabling researchers to classify the biological gender of social media authors based on their language styles in Malay-English code-switching, independently of demographic factors (e.g., age, background or knowledge). Importantly, the accounts selected for scraping met the defined criteria based on minimum post quantity and Malay-English code-switching attributes; also, they were not targeted selectively.

## CRediT authorship contribution statement

**Ruhaila Maskat:** Conceptualization, Methodology, Writing – original draft, Supervision. **Norazmiera Ayunie Azman:** Investigation, Data curation. **Nur Shaheera Shastera Nulizairos:** Investigation, Data curation. **Nurul Athirah Zahidin:** Investigation, Data curation. **Adibah Humairah Mahadi:** Investigation, Data curation. **Siti Rubaya Norshamsul:** Investigation, Data curation. **Mohd Mukhlis Mohd Sharif:** Data curation. **Hairulnizam Mahdin:** Writing – review & editing.

## Data Availability

A Bi-Annotated Malay-English Code-switching Dataset of X posts (Formerly Twitter) for Biological Gender Identification and Authorship Attribution (Original data) (Open Science Framework)

## References

[1] Ikae C., Savoy J. (2022). Gender identification on Twitter. J. Assoc. Inf. Sci. Technol..

[2] Dalyan T., Ayral H., Özdemir Ö. (2022). A comprehensive study of learning approaches for author gender identification. Inf. Technol. Control.

[3] J. Tyo, B. Dhingra, and Z. C. Lipton, “On the state of the art in authorship attribution and authorship verification,” 2022, [Online]. Available: http://arxiv.org/abs/2209.06869

[4] Theophilo A., Giot R., Rocha A. (2023). Authorship attribution of social media messages. IEEE Trans. Comput. Soc. Syst..

[5] Rocha A. (2017).

